# Anal Fistula Human Amniotic Membrane Endosealing (F-HAME): A Proof of Concept Study

**DOI:** 10.3389/fsurg.2022.869923

**Published:** 2022-03-28

**Authors:** Ugo Grossi, Maurizio Romano, Serena Rossi, Gaetano Gallo, Arcangelo Picciariello, Carla Felice, Diletta Trojan, Giulia Montagner, Giacomo Zanus

**Affiliations:** ^1^II Surgery Unit, Regional Hospital Treviso, Treviso, Italy; ^2^Department of Surgery, Oncology and Gastroenterology - DISCOG, University of Padua, Padua, Italy; ^3^Department of Medicine, Surgery and Neurosciences, Unit of General Surgery and Surgical Oncology, University of Siena, Siena, Italy; ^4^Surgical Unit ‘M. Rubino', Department of Emergency and Organ Transplantation, University ‘Aldo Moro of Bari', Bari, Italy; ^5^Department of Medicine - DIMED, University of Padua, Padua, Italy; ^6^Treviso Tissue Bank Foundation, Treviso, Italy

**Keywords:** surgery, fistula, anal, cryptoglandular anal fistula, amniotic membrane (AM)

## Abstract

The treatment of cryptoglandular anal fistula (AF) is often a challenge for surgeons. Several sphincter-saving procedures have been described as an alternative to fistulotomy, with the common goal of promoting healing and preserve anal continence. The aim of this proof of concept study was to assess the outcomes of human amniotic membrane (HAM) implantation in cryptoglandular transphincteric AF. Two consecutive female were recruited. The primary outcome was clinical healing at 6 months. Secondary outcomes were ultrasonographic healing, complications and reinterventions, AF symptoms, fecal incontinence, psychological impact of treatment, recurrence, development of additional AF, patient satisfaction, and quality of life, as measured using validated questionnaires. Both patients (40 and 54-year-old) previously underwent incision and drainage of anal abscess with concomitant seton placement. HAM implantation was performed as a day case under local anesthesia. No intra- or post-procedural complications occurred. Clinical and radiological healing were not achieved at 6 months. However, the external outlet discharge diminished through time, with sustained improvements in quality of life. Clinical healing occurred at 7 months in both patients. Psychological impact of treatment and patient satisfaction were overall good, with improvements in the PHQ-9, GAD-7, and Short Assessment of Patients Satisfaction. HAM implantation is safe and improves patients' quality of life, progressively leading to clinical healing. Future studies are needed to assess its safety in other etiology of AF.

## Introduction

A cryptoglandular anal fistula (AF) is an aberrant, epithelialized tract connecting the inner layer of the anorectum to the perianal skin, with an underlying inflammatory/infective etiology. Its anatomical complexity and the risk of continence impairment represent the main challenges for treatment in many patients. Pain, difficulty in sitting and discharge of pus/blood may be detrimental for quality of life, especially in cases of recurrent disease (1).

Treatment has remained unchanged for centuries, in the form of a fistulotomy (“lay-open”) with knife or cautery, or the use of a seton. However, despite its high healing rates, impaired continence may result from fistulotomy, particularly in patients with high transphincteric AF (2, 3).

Over the last three decades, several sphincter-saving techniques have been described in an attempt to achieve the three main treatment goals, namely closure of the fistula, preservation of sphincter function, and minimization of healing time (4). However, these many available surgical options reflect the ongoing difficulty of achieving lasting fistula closure, with success rates often waning over time (5). Additionally, the heterogeneity of outcomes used in research allied with profound technical variations in surgical management of AF hamper evidence synthesis and meta-analysis (6).

Human amniotic membrane (HAM) is the inner layer of the fetal membranes, with widespread use in clinical practice. Given its antimicrobial, antifibrotic, anti-inflammatory, immunomodulatory, and antiangiogenic properties (7, 8), HAM has gained notable attention for the reconstruction of the ocular surface, for the treatment of diabetic ulcers, as a patch graft for dural repair (9), and in the urology field as treatment for ureteral strictures and vescicovaginal fistulas (10).

In this proof of concept study, we sought to determine the safety and technical feasibility of the HAM in the treatment of cryptoglandular AF.

## Method

### Participants, Assessment, and Outcomes

Two consecutive female patients with primary cryptoglandular transphincteric AF were recruited for this study in July 2021. Exclusion criteria were AF with etiology other than cryptoglandular (e.g., related to Crohn's disease), recurrent AF, presence of >1 external and/or internal orifices, secondary extensions, and concomitant residual abscesses.

Endoanal ultrasonography was performed at baseline (i.e., the day before), 4 and 6 months after HAM placement using a tridimensional (3D) ultrasound device (Flex Focus, endoprobe model 8838; B-K Medical, Herlev, Denmark) by a single colorectal surgeon (UG) with extensive experience in 3D ultrasonography. 3D reconstruction allowed a thorough morphological assessment of AF, reported using a standard template as described elsewhere (11). At the same time points, patients were asked to fill a set of standardized questionnaires, including the Cleveland Clinic Incontinence score (12), the Patient Health Questionnaire-9 (PHQ-9) (13), the Generalized Anxiety Disorder scale (GAD-7) (14), the Short Assessment of Patient Satisfaction (SAPS) (15), and the EuroQol Health Outcome Measure (EQ-5D) (16), with values for Italian patients derived according to Finch et al. (17).

The primary outcome was clinical healing at 6 months, defined as absence of viable external orifices. Secondary outcomes were radiological healing, complications and reinterventions, AF symptoms, fecal incontinence, psychological impact of treatment, recurrence, development of additional fistulas, patient satisfaction, and quality of life.

Approval was obtained from the Ethics Committee of our institution. All procedures were in accordance with the ethical standards of the institutional and national research committee, and with the 1964 Helsinki Declaration and its later amendments.

### Human Amniotic Membrane

The HAM was provided by the Treviso Tissue Bank Foundation. The placenta was source from donors undergoing cesarean sections and processed shortly after retrieval, according to the Italian requirements. The HAM was carefully detached from the chorion and rinsed with sterile saline solution to remove residual blood. It was then flattened on a nitrocellulose membrane filter (Merck Millipore), with its stromal/mesenchymal side in contact with the filter. Afterwards, the HAM was immersed in a cocktail of antibiotics, validated for human tissues, including vancomycin 100 μg/ml, meropenem 200 μg/ml, gentamicin 200 μg/ml at +4°C for 24 h in sterile conditions (18, 19). The HAM was then cut into patches and immersed in cryopreserving solution. Cryopreservation was achieved using a programmable cryogenic freezer (Planer KryoSave Integra, 750-30), which triggered a controlled cooling rate. The HAM patches were then stored in vapor-phase liquid nitrogen. Microbiological analyses was performed at several stages throughout the process. A 6 × 6 cm^2^ patch was used in this study. As shown in previous *in vitro* and preclinical studies, the HAM can yield tissue repair and regeneration based on its immunomodulatory properties.

### Technique

**Figure 1:**
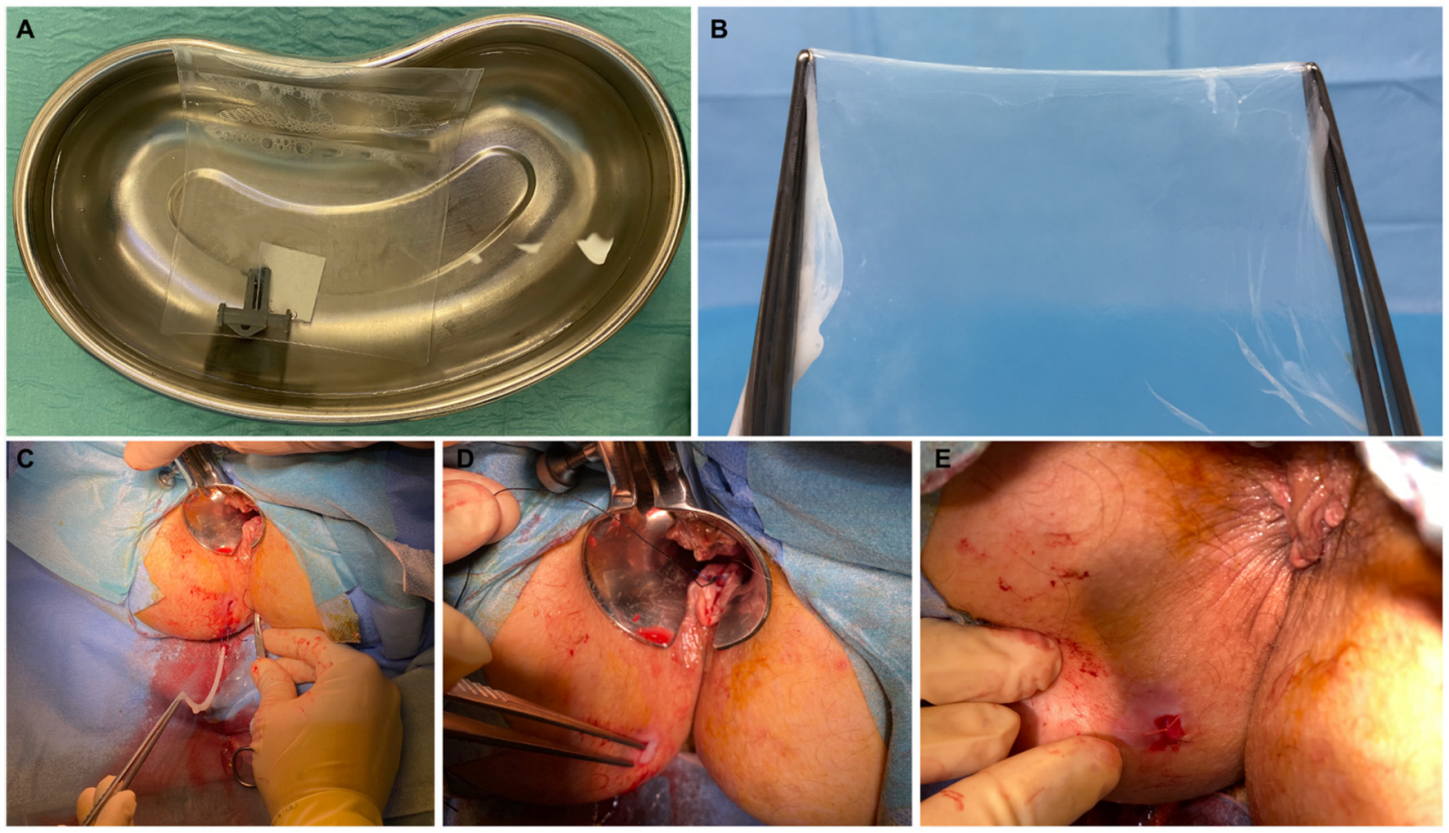
Patient A—A 6 × 6 cm human amniotic membrane (HAM) is defrosted and washed from the cryoprotective medium with hot saline **(A)**; the HAM is removed from the envelope and prepared for implantation **(B)**. The HAM is hooked and pulled through the fistula tract **(C)**. Assessment of the correct placement of the HAM with its ends clearly visible at the level of the external and internal fistulous orifices **(D)**. HAM's distal end fixed to the skin at the external orifice **(E)**.

The procedures were scheduled as day cases under local anesthesia, with water enemas administered before the operation to ensure cleansing of the distal rectal segment. Patients were placed in the lithotomy position. The operative field was prepared in the standard sterile fashion. Perioperative antibiotics were not used. Pudendal nerve block was performed by injection of 10 ml per side of ropivacaine 7.5 mg/ml. After insertion of the Eisenhammer's bivalve retractor, 5–7 ml of bupivacaine 5 mg/ml was injected at the level of the internal orifice. The HAM was defrosted 30 min before use, washed from the cryoprotective medium with hot saline (40°C), and prepared for implantation (Figures 1A,B). These steps were performed under aseptic conditions. The previously placed 4/0 prolene seton was replaced by a 2/0 silk seton. A 4/0 nylon stitch was placed on the vertex of the HAM, fashioning a loop hooked by the distal end of the silk wire, which was pulled through the fistula tract (Figure 1C). This allowed the correct placement of the HAM with its ends being clearly visible at the level of the external and internal fistulous orifices (Figure 1D). The internal orifice was curetted and closed with a Z stitch of 3/0 polyglactin 910. The distal end of the HAM was also fixed to the skin at the external orifice with 3/0 polyglactin 910 (Figure 1E).

## Results

### Participants

Demographics and clinical characteristics of the two female patients are described in Table 1. Both underwent incision and drainage of anal abscess with concomitant placement of a 4/0 prolene seton, 97 and 40 days prior to HAM implantation, respectively. The procedure took between 20 and 25 min, with both patients discharged home on the same day.

**Table 1:** Patients and fistula characteristics.

	**Patient A**	**Patient B**
Age	40	54
No. vaginal deliveries	1	1
American society of anesthesiology score	1	2
No. days since seton placement	97	40
**Primary tract**
Location on a clock dial	Posterior	Anterior
Height	High	Low
Maximum diameter (mm)	1.3	1.4
Location of the internal outlet (hh:mm)	6:00	1:00
Distance of the external outlet from the anal margin (cm)	3.5	2.0
**Anal sphincter morphology**
Internal sphincter	Defect at 6:00	Intact
External sphincter	Intact	Intact

### Outcomes

**Figure 2:**
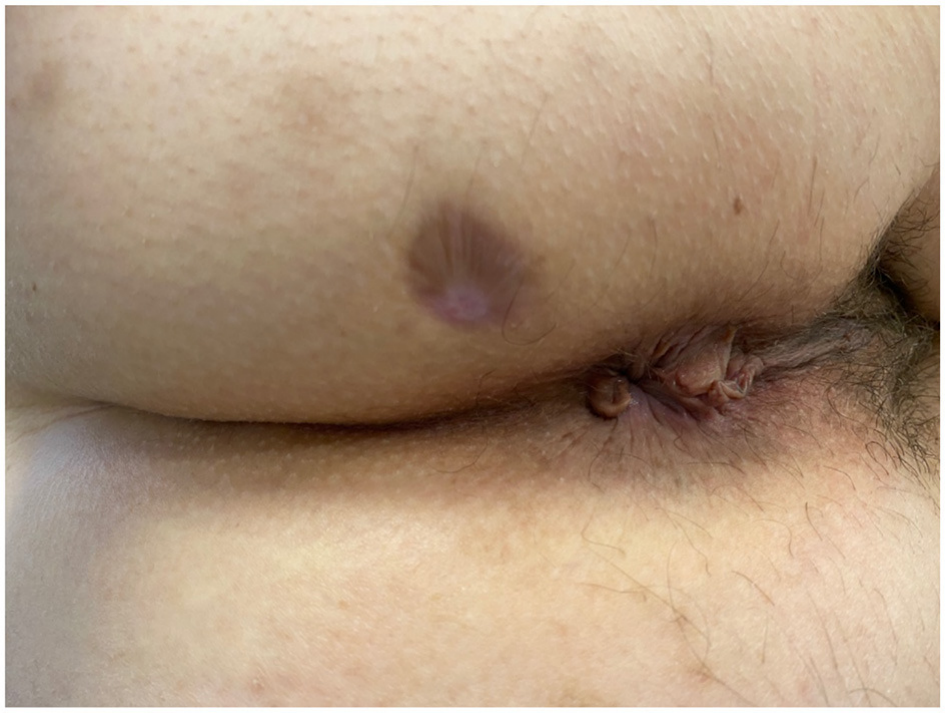
Patient A—View of the perianal area 7 months after HAM implantation. The external orifice is completely scarred and closed.

No intra- or post-procedural complications occurred. None of the patients achieved clinical and radiological healing at 6 months. The external outlet discharge diminished through time, amounting to 4–5 ml per day in both cases. None of the patients experienced fecal incontinence prior the procedure nor this occurred *de novo* post-operatively (Table 2). Endosonographic follow-up did not reveal any development of additional AF. The maximum diameter decreased over 3 months post-implantation in both patients, from 1.3 to 1.0 mm, and from 1.4 to 1.1 mm, respectively. At last follow-up, the fistula tract was not clearly detectable on ultrasonography, which showed only a subcentimetric heterogeneous hypoechoic area in the intersphincteric space. Clinical healing (Figure 2) occurred at 7 months post-implantation in both cases. Psychological impact of treatment and patient satisfaction were overall good, with sustained improvements in the PHQ-9, GAD-7, and SAPS. A similar gain was observed on quality of life, with progressive increasing in EQ-5D values in both patients.

**Table 2:** Secondary outcomes.

	**Patient A**			**Patient B**		
	**Baseline**	**3 months**	**6 months**	**Baseline**	**3 months**	**6 months**
CCIS	0	0	0	0	0	0
PHQ-9	1	0	0	3	1	0
GAD-7	0	0	0	4	2	0
**EQ-5D**
5 points	0.912	1	1	0.784	0.848	1
VAS	70	80	85	75	90	90
SAPS	24	28	28	23	27	28

*CCIS, cleveland clinic incontinence score; PHQ-9, patient health questionnaire-9; GAD-7, generalized anxiety disorder scale; EQ-5D, EuroQol health outcome measure; VAS, visual analog scale; SAPS, short assessment of patient satisfaction*.

## Discussion

Implantation of HAM appears safe in patients with primary cryptoglandular AF. Although clinical healing was achieved more than 6 months post-operatively, psychological impact of treatment was acceptable, with sustained improvements in quality of life.

The presence and volume of discharge, regardless of its content are of utmost importance for patients (20). The gradual reduction in the amount of discharge observed through time, likely mirroring the decrease in maximum AF diameter, may have positively impacted on quality of life. On the other hand, the slow but gradual healing process indicates that a longer follow-up than the canonical 6 months is needed to obtain clinical healing.

HAM has already been applied in multiple sites of the gastrointestinal tract, such as the duodenum, colon, and rectovaginal fistula (21, 22). In a randomized trial, Leila et al. (23) tested the effect of HAM on the wound healing process after anal fistulotomy, with a significant increase in the healing rate observed in the HAM group compared to controls. Its implantation in the AF tract has previously only been attempted in an animal model, associated with endorectal flap, and resulting in improved wound healing (24). To our knowledge, this is the first attempt of HAM implantation in the AF tract in humans.

Recent emphasis has been placed on the potential role of chronic inflammation in the development of AF (25), with epithelial to mesenchymal transition (EMT) representing one possible pathophysiological mechanism both in Crohn's (26) and cryptoglandular AF (27). Future studies are needed to determine whether the immunomodulatory properties of the HAM may interfere with the pathway(s) involved in the proinflammatory process.

Despite reporting on only two highly selected patients, the study was designed in accordance with recent recommendations, by incorporating the newly developed core outcome set, namely the minimum set of outcomes that should be reported in all studies of treatment for cryptoglandular AF (20). Achieving sustained improvements in quality of life may be even more relevant in patients with chronic diseases (e.g., inflammatory bowel diseases).

Although being a proof of concept study, HAM implantation has been well-tolerated under local anesthesia. Given the detrimental impact of the pandemic on national health systems, particularly for benign conditions frequently observed in the proctologic field (28), the implementation of new pathways of care promoting the use of mini-invasive procedures and fast recovery have become common pursuits of healthcare providers and patients. The introduction of a novel sphincter-saving technique often comes with uncertainty regarding its efficacy and whether or not the potential benefits justify the additional cost of the equipment. In this scenario, it is worth mentioning that the HAM implantation is cheaper than other recently developed surgical techniques (e.g., fistula tract laser closure and video-assisted anal fistula treatment).

Future studies are needed to assess the safety and technical feasibility of the HAM implantation in other AF etiology (e.g., Crohn's disease) and in association with alternative modalities of closure of the internal orifice (e.g., advancement flap).

## Data Availability Statement

The original contributions presented in the study are included in the article/supplementary material, further inquiries can be directed to the corresponding author/s.

## Ethics Statement

The studies involving human participants were reviewed and approved by Local Ethical Committee. The patients/participants provided their written informed consent to participate in this study. Written informed consent was obtained from the relevant individuals for the publication of any potentially identifiable images or data included in this article.

## Author Contributions

UG and MR conceived the study. UG, MR, SR, AP, and CF collected the data. UG and GG analyzed the data. All authors interpreted the data and approved the final version of the manuscript.

## Conflict of Interest

The authors declare that the research was conducted in the absence of any commercial or financial relationships that could be construed as a potential conflict of interest.

## Publisher's Note

All claims expressed in this article are solely those of the authors and do not necessarily represent those of their affiliated organizations, or those of the publisher, the editors and the reviewers. Any product that may be evaluated in this article, or claim that may be made by its manufacturer, is not guaranteed or endorsed by the publisher.
